# Deciphering tissue structure and function using spatial transcriptomics

**DOI:** 10.1038/s42003-022-03175-5

**Published:** 2022-03-10

**Authors:** Benjamin L. Walker, Zixuan Cang, Honglei Ren, Eric Bourgain-Chang, Qing Nie

**Affiliations:** 1 The NSF-Simons Center for Multiscale Cell Fate Research, University of California Irvine, Irvine, CA USA; 2 Department of Mathematics, University of California Irvine, Irvine, CA USA; 3 Department of Developmental and Cell Biology, University of California Irvine, Irvine, CA USA

**Keywords:** Computational biology and bioinformatics, Systems biology

## Abstract

The rapid development of spatial transcriptomics (ST) techniques has allowed the measurement of transcriptional levels across many genes together with the spatial positions of cells. This has led to an explosion of interest in computational methods and techniques for harnessing both spatial and transcriptional information in analysis of ST datasets. The wide diversity of approaches in aim, methodology and technology for ST provides great challenges in dissecting cellular functions in spatial contexts. Here, we synthesize and review the key problems in analysis of ST data and methods that are currently applied, while also expanding on open questions and areas of future development.

## Abstract (web summary)

Walker et al. review methods used to analyse spatial transcriptomics data and discuss open questions and areas of future development.

## Introduction

Spatial transcriptomics (ST) methods, in which expression of many genes is measured at a variety of spatial locations in a tissue sample, preserving the source position of each expression datapoint, is a powerful emerging method for understanding functions of cells and their interactions^1–4^. Because the processes by which cells evolve into tissue and communicate with each other depend on interactions with the environment around it, spatial information allows unprecedented insights beyond what may be accomplished by non-spatial single-cell transcriptomic data (i.e., scRNA-seq).

While ST data can be collected in a variety of types and resolutions using different technologies, how to analyze the data to infer spatial organization of cells from discrete datapoints remains a major challenge. Compared to non-spatial data, transforming a spatial biological question to an ST data analysis task, such as uncovering spatial regions of different organized cellular functions from ST data, is often a non-trivial objective whose formulation alone requires more investigation.

Here, we provide a general framework for analyzing spatial transcriptomics data, review the computational methods typically used on ST data (see Table 1 for concise list), and overview the resulting analyses that can be performed. We highlight the considerations and limitations of these methods, and discuss the intriguing future areas for development in this field.

**Table 1:** List of software packages.

Name	Summary	Platform	Reference
Identifying spatially variable genes			
Trendsceek	Statistical testing on spatial hypothesis (non-parametric)	R	^25^
SpatialDE	Gaussian process regression	Python	^26^
SPARK	Statistical testing - generalized linear spatial model	R	^27^
SOMDE	Self-organizing neural map + Gaussian process regression	Python	^28^
Sepal	Assessing spatial variance by length of time to equalize under diffusion	Python	^29^
scGCO	Graph cuts to divide based on spatial expression	Python	^30^
SpaGCN	Graph convolutional network, joint detection of regions	Python	^31^
Region Segmentation			
stLearn	Histology-based smoothing + clustering	Python	^21^
Seurat	Non-spatial clustering combined with spatial visualization	R	^22^
SmfishHmrf (Giotto)	Combining Gaussian expression model with hidden Markov random field	R	^23,33^
SpaGCN	Graph convolutional network, joint detection of SVGs	Python	^31^
BayesSpace	Fully Bayesian expression model, hyper-resolution segmentation	R	^34^
SEDR	Deep auto-encoder based embedding for clustering	R	^35^
Identifying cell-cell interactions			
SpaOTsc	Optimal transport to match ligand and receptor expression	Python	^41^
Spatial Variance Component Analysis	Gaussian process model including interaction term	Python	^42^
Misty	Multi-component linear model including interaction term, random forest	R	^43^
Node-centric Expression Modeling	Graph neural network combining expression data over various length scales	Python	^44^
GCNG	Supervised training of graph neural network then allows for identification of novel interactions	Python	^45^
Mapping cells to spatial locations			
Seurat	Alignment for a variety of data modalities including spatial data by pairing a subset of cells as anchors	R	^22^
SpaOTsc	Optimal transport mapping between spatial and single cell data	Python	^41^
DistMap	Matthews correlation coefficient computed on binarized expression	R	^53^
DeepSC	Neural network learns to predict locations of cells in space	Python	^54^
GLISS	Uses graph-based measure based on similarity of landmark genes	Python	^55^
Tangram	Aligns gene expression while also accounting for spatial cell density distribution	Python	^56^
Cell type deconvolution/enrichment scores			
Giotto	Several algorithms for computing enrichment scores	R	^23^
SPOTLight	Non-negative matrix factorization using known marker genes for initialization	R	^49^
SpatialDWLS	Dampened weighted least squares for matrix factorization	R	^50^
RCTD	Statistical fitting of combination of Poisson distribution models	R	^51^
DSTG	Graph neural network to learn cell types and deconvolution from data	Python	^52^

## Overview of spatial transcriptomics data

### Data collection methods

Current methods for collecting spatial transcriptomics data include spatial barcoding, in which the barcodes used in identifying RNA molecules are coded to indicate location; and fluorescent hybridization, in which RNA molecules are tagged with a fluorescent compound and then captured using single-molecule imaging; in situ-sequencing based methods; and dissection methods, where tissue is divided into sections which are then sequenced with non-spatial methods similar to RNA-seq.

Spatial barcoding procedures place barcodes containing information that allows RNA captured within to be tied to the original spatial location on a slide, and a slice of tissue is placed onto the slide such that RNA from cells is tagged with the spatial barcodes^4,5^. These spatial barcodes may be constructed as part of a regular grid of spots, or through randomly deposited beads. Successive techniques seek to construct spatial barcodes in which the original position can be localized with increasingly fine resolution, in addition to increasing gene coverage and capture efficiency. The Visium technology^6^ captures gene expression using an array of approximately 5000 spots with diameter 55 μm. In addition to the expression data for each spot, a stained image of the tissue is captured. Slide-seq^7,8^ uses beads randomly deposited on a puck, with a 10 μm spatial resolution. High-definition Spatial Transcriptomics (HDST) captures at a ~ 2 μm resolution^9^ and Seq-scope^10^ further increases the resolution with a center-to-center distance of ~ 1 μm.

Fluorescence in situ hybridization (FISH) methods rely on fluorescence to identify specific RNA molecules with high-resolution optical imaging. In these methods, RNA molecules are hybridized and then the resulting fluorescent colors are measured through imaging to identify and localize RNA molecules. By encoding a particular RNA species as a sequence of colors, and then tagging that RNA with the respective colors in successive rounds of imaging, a number of RNA types exponential in the number of rounds can be distinguished. Then, the RNA molecules are divided by the cell they originate from to produce a cell-by-count matrix spatially indexed by the centroid position of each cell. This contrasts with spatial barcoding methods in which spatial locations do not directly correspond to individual cells. Multiplexed Error-Robust FISH (MERFISH)^11^ uses error coding to increase accuracy in measurement and can measure over 10,000 genes. seqFISH+^12,13^ uses a larger number of colors to reduce the number of imaging rounds required for data collection and is capable of measuring counts for up to 24,000 genes. Compared to spatial barcoding methods, FISH datasets tend to capture a lower number of genes but allow for accurate localization of individual RNA molecules and significantly higher capture efficiency^5^, giving a more accurate picture of the genes that are captured.

Additional methods for the collection of ST data include in situ sequencing methods, in which RNA molecules are reverse transcribed into DNA and then sequenced within the cell, such as FISSEQ (Fluorescent In Situ Sequencing)^14^, BaristaSeq^15^, and STARmap^16^. Alternately, technologies such as Geo-seq^17^ and Tomo-seq^18^, which use cryosectioning, separate tissue into small sections and then perform RNA sequencing. This allows for the final collection to be done using non-spatial sequencing, allowing for higher capture efficiency, but require increased preparation of the sample and are therefore significantly limited in both the number of spatial locations that are extracted and the resolution at which they are separated.

### Multiple resolution scales in spatial transcriptomics data

**Fig. 1:**
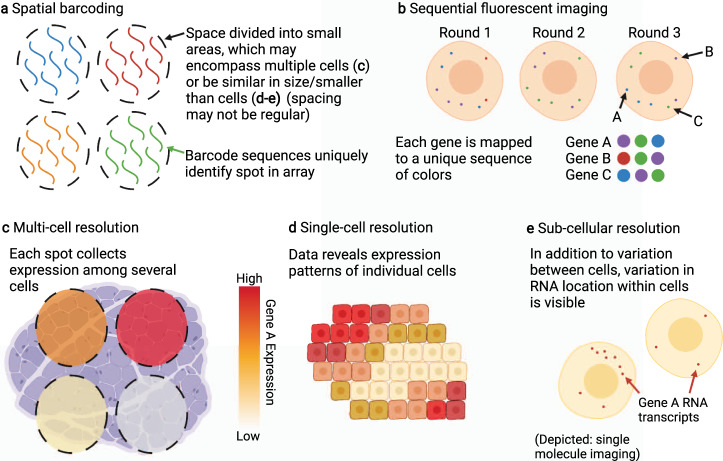
Spatial Transcriptomics Data: Collection and Resolutions. ST data can be collected with various methods and resolutions. **a** Illustration of spatial barcoding, in which spatially-identified barcodes are arranged and then used to tag RNA molecules in tissue. Compare with **c**, but note these methods are not restricted to multi-cell resolution. **b** Illustration of sequential fluorescent imaging, where RNA molecules are sequentially tagged with different color fluorescent probes and the color sequences are used to identify RNA species. In general, this data is collected at sub-cellular resolution, as show in **e**, but is frequently combined with cell segmentation to create single-cell data, as in **d**. **c** Multi-cell resolution spots, in which measured expression at one spatial location is collected across a number of possibly heterogeneous cells. **d** In single-cell resolution data, each spatial location corresponds to one cell. This allows for spatial analysis of cell identity and a single-cell understanding of tissue structure and cell-cell communications. **e** One type of sub-cellular resolution data is single-molecule imaging. Note the presence of information both in the number of distinct RNA molecules of one type in a cell, and also the localization of those molecules within the cell. Sub-cellular resolution data may be combined with cell segmentation to produce single-cell data to facilitate corresponding analysis.

Currently, there are three major scales in ST data: multi-cell, single-cell, and sub-cellular resolution. In multi-cell resolution data (Fig. 1a) each spatial datapoint may contain genetic material from multiple cells of varying number and type. As such, downstream analysis typically considers expression as a combination of contributions from multiple cell types in a manner similar to bulk RNA-seq analysis, but on a much smaller scale. Single-cell resolution data (Fig. 1b) is characterized by locations that are either exactly single cells or spots on the scale of a single-cell. Sub-cellular resolution (Fig. 1c) data localizes the positions of RNA molecules on a spatial scale smaller than the size of a cell. This may take the form of high-resolution spatial barcoding, where spots are smaller than single cells, or single-molecule imaging where positions of individual RNA molecules are captured. Sub-cellular resolution data can also be combined with cell segmentation to produce single-cell resolution data where expression is tied to specific cells, which can be processed in spatially-aware single-cell analysis pipelines.

Some downstream analysis tasks may be associated with data of a specific resolution. For example, cell type decomposition analysis is applied to multi-cell resolution data to decompose the expression into percentage contributions from different cells or cell types. Alternately, sub-cellular resolution ST data can be extended beyond analysis of count matrices to also consider the position of specific RNA species within the cell^19^, using the position of RNA molecules inside the cell to provide information on cell type and state beyond that of summed counts. This additional information may allow for more detailed or precise discrimination of cells, but at a tradeoff of increased data complexity.

## Preprocessing of ST data

As in the case of scRNA-seq data, preprocessing is an essential step in the analysis pipeline. In general, as ST data typically consists of a collection of transcriptomic barcodes, the same practices as used in scRNA-seq analysis may be directly applied to ST data. Standard practices include the steps of filtering out low-quality barcodes, normalizing counts, and controlling for undesired experimental effects or covariates^20^.

However, ST data includes positional information, and this data can also be leveraged in the preprocessing step, under the assumption that nearby cells are more likely to be similar expression. The stLearn method^21^ performs smoothing on the expression count data over the spatial neighborhood of a spot, using Visium data. To account for the possibility of nearby but dissimilar cells, the neighborhood smoothing is weighted by a morphological similarity score, derived from application of a pre-trained convolutional neural network to an image of each spot, more heavily weighing neighbors that are deemed morphologically similar.

In principle, the use of smoothing techniques is helpful in improving the quality of downstream spatial analyses. For example, as this step seeks to average out expression profiles from nearby cells of the same type, an iterative procedure could use downstream computation of cell types in the spatial smoothing step. More advanced techniques for in-depth exploration of the spatial information at the stage of pre-processing are needed.

In order to facilitate pre-processing of data and serve as a computational framework for downstream analysis, a number of packages have been introduced for processing transcriptional data with spatial information, such as Seurat^22^, Giotto^23^, Squidpy^24^, and stLearn^21^.

## Defining and identifying spatially variable genes

A key step in scRNA-seq pipelines is the identification of highly-variable genes (HVGs), for which expression exhibits significant differences between cells. However, a gene may exhibit variation from cell-to-cell but not in a way that produces a clear spatial pattern when viewed using ST data. As such, in order to understand spatial cellular variation, analysis of ST data requires the identification of spatially variable genes (SVGs). These spatial variations in gene expression can reflect cell type compositions that perform specific spatial functions or spatial patterns in cell-cell interactions^23^. Spatial expression of SVGs may exhibit patterns such as clustering and periodicity, depending on the tissue structure and function. Methods for detecting spatially-variable genes can be mathematically understood as expressing the cell-to-cell variation exhibited in gene expression as a combination of spatial variation, which occurs on a coherent pattern in space, and non-spatial variation, including intrinsic variation between cells and possibly other terms, such as variation due to cell-cell interaction (see Supplementary Note 1, *Spatially-variable genes*). When the variation of a particular gene is primarily due to spatial variation, that gene can be said to be spatially variable.

A variety of recent methods have been proposed that vary in the manner in which spatial variance is represented. Trendsceek^25^, SpatialDE^26^ and SPARK^27^ are methods based on spatial correlation testing, where the correlation between the distribution of gene spatial expression and the data site locations is considered. SpatialDE models the variability of gene expression using Gaussian Process Regression, where the expression variability is decomposed into spatial and non-spatial parts^26^. The spatial covariance between cells is modeled to decay exponentially with the squared distance between them, and comparison of the spatial and non-spatial contributions of variance provides a natural and interpretable explanation of spatial patterns in genes. However, it is limited by the choice of kernels used in the Gaussian process model. Trendsceek instead assesses the relationship between gene expression levels and spatial locations non-parametrically^25^, modeling expression as a marked point process. Testing for significance with a permutation-based test, non-linear expression patterns can be identified without the need to specify a distribution or spatial region of interest. This, however, comes at the cost of significantly increased computation time. SPARK (Spatial PAttern Recognition via Kernels) directly models spatial count data through generalized linear spatial models (GLSM), a mixture containing both periodic and Gaussian kernels to directly model the non-Gaussian spatial data, and uses random effects to capture the underlying stationary spatial process^27^. The computational complexity of the above methods grows quadratically as the number of spatial sites increases, and so they may be difficult to apply to larger datasets. SOMDE^28^ instead uses a self-organizing map neural network model to combine cells into nodes that preserve the expressional and topological structure of the data, effectively a coarse-graining step, and then applies a Gaussian process model similar to SpatialDE.

Based on the mathematical principle that non-spatial variation will correspond to higher-frequency modes in space, and spatially significant variation to lower-frequency, Sepal^29^ performs a Gaussian diffusion on the spatial expression. Because higher-frequency variation decays exponentially faster under diffusion, the timescale on which spatial variation persists through diffusion is indicative of the significance of spatial structure in a gene. Mathematically, this can be understood as representing variation on a continuous scale between spatial and non-spatial. scGCO (single-cell graph cuts optimization) applies a graph cut method analogous to those used in image segmentation^30^. scGCO applies a Delaunay triangulation across tissue to generate a sparse graph representation of data sites and then adopt binary cuts on the graph via optimization of Markov Random Fields. SpaGCN identifies spatial domains and SVGs jointly^31^ by using a graph convolutional neural network to learn a representation aggregating gene expression data from surrounding spots. The adjacency graph used in the convolution is constructed based on both spatial location and histology, which enables identification of SVGs and domains with coherent expression and histology.

Compared to other analyses, it is much more difficult to quantify what exactly constitutes a spatial pattern, despite how obvious it is to the human eye. Consequently, when identifying genes with interesting patterns in a dataset is important, it may be particularly useful to apply multiple methods with differing approaches, which may have the capacity to recover different types of patterns.

## Segmentation of spatial regions with distinct biological functions

While clustering cells into groups with similar expression is a common task in scRNA-seq analysis, spatial data allows for the much more powerful segmentation of data into distinct spatial regions. Cells contribute to various biological functions when cooperating with other nearby cells, and using spatial transcriptomics data, we can identify these spatially associated groups to understand how different cells work together to perform complex functions. This leads to the task of dissecting the tissue into spatial domains. Depending on the type and resolution of data, the spatial locations that are being segmented into regions may be, for example, individual cells or spots in a spatial barcoding array, but below we will refer to any such single location in a spatial transcriptomics dataset as a spot for brevity.

**Fig. 2:**
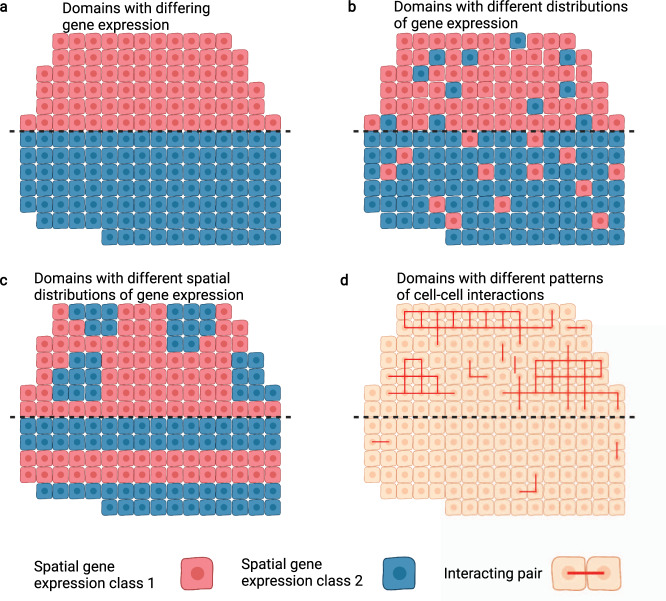
Illustration of different traits that can separate spatial regions. **a**–**d** Dotted line indicates division between two regions. Red and blue cells indicate groups with consistent expression across some set of spatially variable genes. **a** Regions are characterized by different gene expression, equivalent to the groups identified by cluster analysis such as the Louvain algorithm on non-spatial data. **b** Regions are not entirely homogeneous, but instead differ in distribution of observed expression. **c** Regions have similar distributions, but differ in the spatial patterning of gene expression. **d** Red lines connect interacting cells. Beyond cell type indicated by gene expression, regions may be distinguished by higher-level properties such as patterns of cell-cell interactions. Performing region identification downstream of other analyses could allow for detecting variance in such properties.

Before developing computational tools for identifying these domains, it is necessary to define what constitutes a domain in the first place. Segmentation can be loosely viewed as an optimization problem, attempting to group spots into maximally similar spatial regions under some objective defining similarity (see Supplementary Note 1, *Segmentation*). The simplest approach is to look for spatially contiguous regions of cells with maximally similar gene expression (Fig. 2a). This is analogous to the typical clustering analysis in scRNA-seq analysis pipelines, but conscious of spatial position. However, if viewing regions from a functional perspective, they may not simply consist of a homogeneous collection of cells with similar gene expression. Other ways of defining a spatial domain lead to different interpretations which are still underexplored. For example, regions might consist of heterogeneous collections of cells with differing gene expression, but distributed such that there are not clear sub-regions (Fig. 2b). Regions might also be defined by the particular arrangement of cell types (e.g., salt and pepper versus layers, Fig. 2c), or may be distinguished in terms of morphological features, revealing functions associated with morphological characteristics. Note that while the regions depicted in Fig. 2c could be divided into meaningful sub-regions, other aspects beyond simple transcriptional similarity could also reveal function differences between spatial regions—for example, spatial domains associated with functions regulated by cell–cell communications (Fig. 2d) could be identified by performing domain segmentation downstream of cell–cell communication inference. However, current approaches for identifying spatial regions primarily center on the first definition, identifying spatially nearby groups of cells with maximal similarity in gene expression.

One approach to identifying regions in spatial data is to apply standard clustering techniques used in scRNA-seq analysis, such as the clustering functionality in Seurat^22^. This allows for some visualization of spatial clusters; however, without incorporating spatial information, the full potential of the data is not used. This can be improved with a pre-processing step that incorporates spatial information into the similarity used in the clustering algorithm. For example, spatialLIBD^32^ first identifies specifically genes exhibiting layer-based variation in expression in a human prefrontal cortex dataset, and then performs clustering analysis only using those SVGs. In this way, output represents functional layers as opposed to simply cell types. stLearn^21^ applies a pre-processing step in which expression data is smoothed based on morphological similarity between cells as determined using a pre-trained computer vision algorithm trained on image classification tasks, applied on staining images which are often available as byproduct of spatial transcriptomics. This increases the similarity between morphologically similar locations, and as a consequence after performing clustering morphologically similar regions of the tissue will be more likely to be associated into a domain. In order to ensure spatial contiguity of domains, after the clustering step any disconnected clusters are split into subclusters representing contiguous regions. While morphological similarity measures are highly interesting in the analysis of spatial data, given the relative ease of access compared to other alternate data modalities, there remains significant room for future research into the development of automated image analysis tools tuned specifically on histology images and designed to integrate with downstream ST data analyses.

In addition to modifying and adapting preprocessing steps in scRNA-seq data analysis for spatial transcriptomics data analysis, models can be designed natively for spatial data at the clustering step, removing the need for post-processing to ensure domains are connected in space. A hidden Markov random field (HMRF) method, SmfishHmrf^33^, also included in Giotto^23^, combines a Gaussian model of gene expression with a spatial term that explicitly incentivizes cells that are adjacent in the proximity graph to be part of the same region. Using an expectation-maximization algorithm, optimization is simultaneously performed over the type of each cell and the expression pattern of each cell type. The HMRF method produces regions that are contiguous in space, but is limited by the simple Gaussian model used for expression and has a tendency to create blocky regions without complex boundaries. However, the formulation easily lends itself to adaptation with other segmentation objectives (e.g., as shown in Fig. 2b–d) so it may be useful in future methods development. BayesSpace^34^ uses a more powerful, fully Bayesian formulation to model spatial region-based gene expression, using a Markov random field prior to create spatial coherence. Of particular note in the BayesSpace method is an additional part of the algorithm in which each spot in the array is subdivided into subspots whose expression levels are learned to allow the subspots to fit into different regions, constrained by the original spot-level expression. This approach allows for results to be projected on a resolution higher than the data was originally collected at. However, the structuring of the Bayesian approach is specific to the arrangement of spots, and is therefore more difficult to adapt to non-Visium data.

Recent work has also sought to apply machine learning methods to learn how to separate cells or spots into regions. As mentioned previously, SpaGCN^31^ detects both SVGs and spatial regions jointly through application of a graph convolutional network. Spatially Embedded Deep Representation (SEDR) constructs an embedding that jointly captures expression and spatial information through a deep autoencoder framework^35^. Such deep learning embedding methods offer increased discriminatory power through the more complex model, but can suffer from lack of interpretability in the resulting embeddings. However, the high-information embedding can be leveraged for additional downstream analyses such as trajectory inference and batch correction^35^.

## Cell-cell interactions in space

**Fig. 3:**
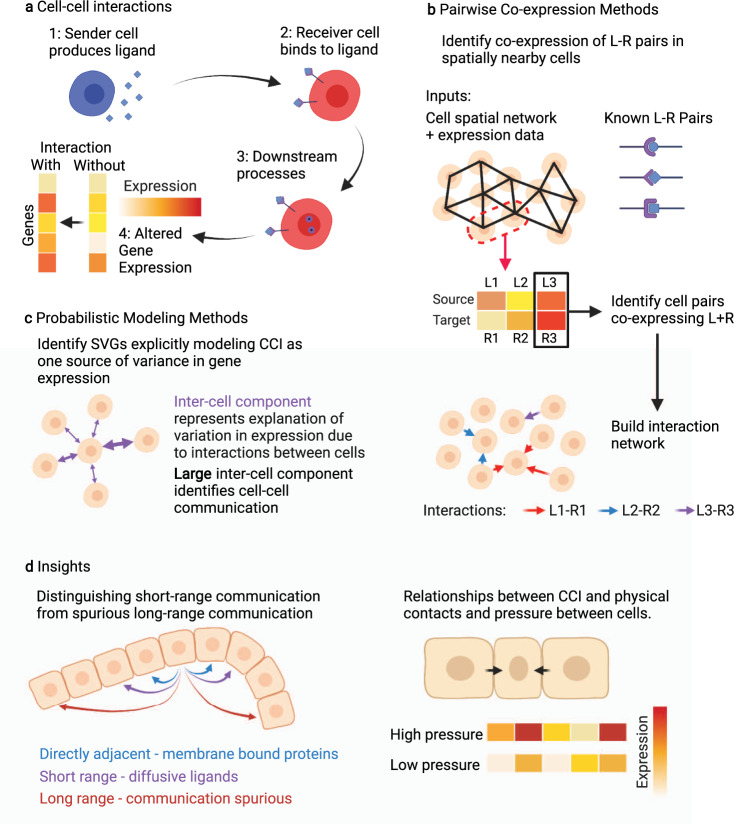
Illustration of techniques in extracting cell-cell interactions from ST data. **a** Cell-cell interactions occur when transfer of a ligand from a sender cell to a receiver cell triggers a downstream response, ultimately leading to changes in gene expression in the receiver cell. **b** Common techniques identify co-expression of known L-R pairs in cells adjacent in a spatial proximity network, and use this to mark interactions between cells. **c** Alternatively, some methods probabilistically capture different sources explaining variance in spatial gene expression, including terms capturing intra- and inter-cellular effects. When inter-cellular effects dominate a particular gene’s expression, it is indicative of cell-cell interaction. **d** Insights made from CCI analysis of spatial data include the ability to determine interactions of a particular cell by filtering out spurious long-range connections, and investigations into the relationship between L-R interactions, and mechanistic interactions and cell proximity.

In most tissues, the interaction and communication among cells happen at a short timescale compared to cell movement and migration. Given the relative stability of cellular locations, spatial transcriptomics allows us to reveal cell–cell interactions (CCI), also referred to as cell-cell communications (CCC), with fewer false positives than similar analysis with scRNA-seq data. Analysis of interactions between cells can be divided into two sections: identifying pairs of genes that interact, such that expression of the gene in one cell influences that of the other gene in others (Fig. 3a); and identifying pairs of cells in which that gene pair interacts. Here we will discuss methods that identify pairs of interacting genes from ST data, as well as those that use prior knowledge of interacting genes to identify interactions between cells or groups of cells.

Ligand-receptor (L-R) interactions follow chemical pathways whose existence is not specific to any one organism, and often not even any one species, and as such, identification of CCI can benefit particularly heavily from borrowing previous knowledge, typically in the form of curated L-R databases whose entries correspond to known interactions that have been established in prior literature^36–39^. Given such a database, inference of cell–cell communications can be naturally extended to the spatial case using an approach that looks for pairs of cells that co-express a particular known L-R pair and are also sufficiently nearby in space^21,40^, as shown in Fig. 3b. In this way, interactions between cells that would be presumed when only considering L-R co-expression can be filtered out if there is not a physical possibility for communication between them (Fig. 3d). SpaOTsc^41^ uses an optimal transport method to match ligand and receptor distributions to create a cell-level map of which cells communicate with which other cells.

Similar to methods for identifying spatially variable genes, statistical models of gene expression in space that model multiple genes simultaneously can identify pairs of genes whose interaction explains large amounts of variance and thereby extract interacting genes (Fig. 3c). These methods allow for the discovery of novel interactions supported by the data, which is not possible when only using L-R interactions from databases. Spatial Variance Component Analysis (SVCA)^42^ uses a Gaussian process model where the covariance matrix has a term modeling interaction between cells, and if this term is large compared to other covariance terms representing intrinsic variation and random noise, the cells are considered to be interacting. Misty^43^ uses a similar multi-component model using a random forest machine learning framework to learn the different components in the model. Node-centric expression modeling (NCEM)^44^ uses a graph neural network model on varying length scales, allowing it to learn higher-order interactions and determine characteristic spatial scales of interaction. This attention to identifying from data not only interactions but also length scales is particularly interesting and highlights a key benefit of analysis of CCI on ST data over scRNA-seq data. GCNG^45^ uses supervised machine learning on known interacting pairs to produce a model that can then identify novel pairs from ST data. However, the supervised training approach may be particularly sensitive to the choice of data originally used to train the model.

In addition to inferring the communications among cells based on known ligand-receptor pairs, spatial transcriptomics also allows for more detailed study of the interplay between spatial arrangement and CCC. For example, identifying adjacent cells from spatial data can reveal CCC through membrane-bound proteins, which lack the longer-range diffusion of other communication methods^23^. Beyond just identification of interactions between cells, inferences can further be made into the role that ligand-receptor interactions play in higher-order tasks such as the spatial arrangement of cells^40^.

## Determining spatial distribution of cell types in multi-cell resolution data

Widely used ST techniques such as the Visium technology^6^ collect data at a spatial resolution that often corresponds to 2–8 cells. In order to understand spatial tissue structure in terms of single cells, the ST data can be augmented with cell type information either from a provided atlas or in an unsupervised manner from scRNA-seq data through standard clustering analysis such as with the Louvain algorithm^46^.

**Fig. 4:**
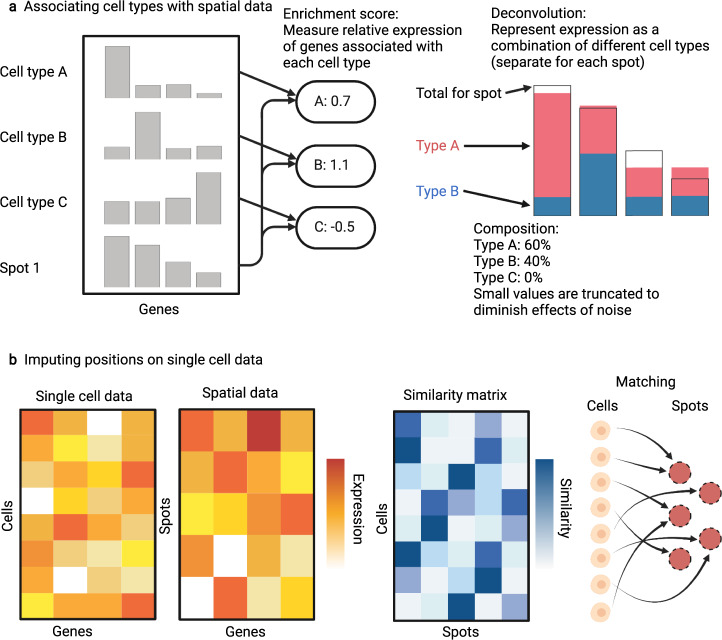
Enhancing spatial transcriptomics data with scRNA-seq data. This analysis step augments ST data using scRNA-seq data. **a** By using scRNA-seq data onto the spatial dataset, the composition of individual spots can be understood in terms of single cells, such as by computing enrichment scores, which measure the expression of certain gene sets (such as marker genes from a particular cell type) relative to the norm, or through deconvolution, which decomposes the overall expression data from a spatial spot into a combination of contributions from several cell types. **b** scRNA-seq data can be used to increase resolution of multi-cell ST data, by mapping cells to spatial locations, producing a spatial dataset at single-cell resolution. The primary choices in such methods are the computation of similarity scores between cells and spots, and the method by which matching is computed from the similarity matrix.

One way to quantify the presence of cell types at each spot is to compute enrichment scores, which represent the relative expression level of some set of genes. By identifying a set of marker genes for a particular cell type, the enrichment score of that gene set at some spot is informative of the presence of that cell type at that spot (Fig. 4a). The Seurat package^22^ allows for computation of enrichment scores through the AddModuleScore function. The Giotto package^23^ computes enrichment scores in three ways: using the PAGE algorithm^47^ in which a normal-distribution based statistical test is used to assess significance; an algorithm that uses gene expression rankings to avoid the need to compute explicit sets of marker genes, and a hypergeometric test on an expression contingency table. Multimodal Intersection Analysis (MIA) computes an enrichment score for cell types over spatial regions by identifying marker genes for each cell type and each spatial region, and measuring the extent of overlap between corresponding sets of marker genes^48^.

An alternative to enrichment scores is deconvolution analysis, in which the total expression at each spot is broken down into a percentage decomposition of different cell types (see Supplementary Note 1, *Deconvolution analysis*). Compared to enrichment scores, this decomposition is more directly interpretable and allows for cell types to be clearly mapped to different regions in space. A non-negative matrix factorization regression^7^ approximates the expression matrix as the product of a (non-negative) coefficient matrix and the expression pattern of known cell types from scRNA-seq data. This coefficient matrix can then be interpreted as a decomposition of each spot in terms of cell types. SPOTLight^49^ extends the non-negative matrix factorization approach by using known marker genes to initialize the factor matrices, improving stability over the base NMF method. SpatialDWLS^50^ similarly uses a Dampened Weighted Least Squares algorithm, originally designed for bulk RNA-seq data to decompose expression at spots into individual cell types, obtaining higher accuracy than NMF methods. Robust Cell-Type Decomposition (RCTD)^51^ fits a statistical model in which gene counts are assumed to be Poisson distributed to infer a decomposition of cell types at each spot, and directly accounts for the effects of experimental differences between the spatial data and the single-cell data. Given that there are likely to be significant differences on how data was collected between the spatial and single-cell datasets, correcting for batch effects, a highly studied phenomenon in the literature, is a natural and valuable step to add to this process. DSTG^52^, a machine-learning based alternative, uses graph convolutional networks to predict cell type compositions. In this approach, scRNA-seq data is used to create a pseudo-ST dataset, in which spots are generated by randomly combining expression data of cells. This pseudo-ST dataset is used to train a graph convolutional network that predicts cell type decompositions in the real ST data. However, there remains room for deeper investigation into how to construct such pseudo-ST datasets for training machine-learning based approaches and perform more detailed comparisons between machine learning and more traditional approaches.

## Utilizing scRNA-seq data to improve resolution of spatial transcriptomics data

When analyzing multi-cell resolution ST data, scRNA-seq data can also be used to produce a finer, single-cell resolution spatial dataset by relating single cells to spatial positions through a similarity measure between the ST data and the scRNA-seq data, as illustrated in Fig. 4b. These techniques allow for the analysis pipelines from single-cell resolution spatial transcriptomics to be extended to spatial data collected from spatial barcoding methods. In this case, each cell in an scRNA-seq dataset is matched to a location by comparing expression data between the scRNA-seq and the spatial transcriptomics data. Typically, the expression data of each cell is compared to each spot and a similarity score is computed, possibly in some shared latent space, combined with a statistical test for significance. Mapping to the latent space can be viewed as a dimensionality reduction problem, and can also be used to address other issues such as batch effects and technical noise. The DistMap algorithm^53^ binarizes gene expression and then scores the similarity between cells and spots using the Matthews correlation coefficient. The SpaOTsc method^41^ poses the problem as matching two distributions of cells over transcriptional space and applies a structured optimal transport algorithm to find a matching between cells and locations that maximizes similarity between the expression data of the cells and that at their imputed location, while ensuring cells are properly distributed over the area. Seurat^22^ includes a method that projects spatial and scRNA-seq datasets to a shared latent space using canonical correlation analysis, scoring similar cells by shared neighborhood and distance in that space. DeepSC^54^ uses a deep-learning method to learn an adaptive metric representing the probability of a given cell occurring at a particular location given the respective expression data of each, and then matches cells to their most likely originating location. GLISS^55^ is a graph-based method that uses a Laplacian Score to identify landmark genes, constructs a graph based on similarity of gene expression among landmark genes, as well as between landmark genes and SV genes. Tangram^56^ maps single-cell data to spatial data probabilistically by minimizing KL divergence of cell density at spatial regions while also accounting for correlations between gene expression of single cell and spatial data and spatial origins.

For the integration methods depending on gene expression similarities, the constructions of the correlation or similarity matrices between scRNA-seq and spatial data is crucial to the mapping quality. The aforementioned methods introduce different criteria for selecting genes to use in the mapping, as blindly using all common genes or simply non-spatial HVGs may cause contamination of the connectivity matrix from spatially unmeaningful genes. Differences in construction of the similarity or correlation metric can also significantly affect results. Generally, a sparser connectivity matrix leads to more precise but less robust mappings. Another challenge is that the gene expression levels between scRNA-seq and spatial transcriptomics are not linearly related in practice, and therefore methods using similarity measurements based on ranking or binarized values may produce more robust results. Furthermore, a potential issue of high-resolution mapping between scRNA-seq and ST data is that individual cell-spot pairs may be less reliable, as higher spatial resolution typically leads to fewer counts, similar to scRNA-seq data analysis in which observations of groups of cells are more generally reliable than those of individual cells. To tackle this issue, the cluster-level mapping between scRNA-seq and ST data discussed in the section *Determining spatial distribution of cell types in multi-cell resolution data* may be used as a measurement of confidence of the individual cell-spot level mapping.

## Conclusions and future outlook

Recent advances in spatial transcriptomics technologies allowing higher resolution, greater gene coverage, and lower experimental cost have sparked an explosion in methods for analyzing the resulting data. These advances thus drive the growth of computational methods and pipelines for ST data analysis, allowing deeper discovery of biological insights. In this review, we have surveyed the primary types of analysis that are performed along with current methods and software, highlighting their variations in suitability for different datasets and in outcomes.

There remain a number of promising avenues of research in future development of computational tools, which will lead to more extensive and rigorous analysis of ST datasets, allowing deeper discovery of biological insights. While new methods for performing region segmentation continue to be developed at a significant pace, current methods center on the same notion of similarity from scRNA-seq, looking for groups of cells with maximally similar gene expression, but constrained to exhibit spatial coherence. Because functional regions of tissues may not be composed entirely of cells with identical expression patterns (as shown in Fig. 2b–d), this limits the potential to detect meaningful regions in ST data. Recent work on deep generative models^44,57^ for modeling gene expression suggests the possibility of capturing more complex expression patterns in each region than a simple maximization of transcriptional similarity. Additionally, as regions consist of multiple cells, the native inclusion of multi-cell properties such as cell-cell interactions will enhance the ability of region segmentation methods to understand the structure and function of the tissue.

There is also significant room for the development of improved and accessible tools for inferring cell–cell communications from spatial data. For example, the use of spatial data allows for informed predictions to be made about potential communications between individual cells, instead of analysis between groups of cells that is typical in scRNA-seq data, and this may be a focus in future methods. While gene regulation and ligand-receptor interaction are major mechanisms by which cells interact, there are also other aspects in cell-cell interactions that may come to light using spatial data such as downstream reactions caused by mechanical pressure and physical contact between cells (Fig. 3d). This analysis, previously intractable, is now possible by analyzing the morphological characteristics of cells with the detailed cell shapes revealed by imaging combined with ST data. Alternately, a recent technology, PIC-seq^58^, isolates and sequences pairs of cells in physical contact, providing a different type of spatial information than traditional ST data, which could be combined with traditional ST data to improve analysis of physical relations between cells. As validation is a challenging problem in inference of cell–cell communications from both spatial and non-spatial data, these new data modalities and corresponding computational analyses present an opportunity for a clearer and more robust picture of cell–cell communications.

Another notable avenue for improved development of spatial algorithms is pseudotime analysis, which has been used extensively on scRNA-seq data to understand phenomena such as cell differentiation and cancer progression. Traditionally, cell state trajectories are built from single-cell expression snapshots, where the spatial structure within a tissue is largely ignored. This can hinder our discovery and understanding of the dynamics of progression on the tissue level. Recently, stLearn^21^ has proposed a concept of pseudo-space-time (PST), calculated by taking a linear combination of non-spatial diffusion pseudotime and spatial distance. Whereas pseudotime values computed from a particular root cell represent distance along the manifold of gene expression taken by cells through development, a measure of spatial pseudotime should represent a combined distance in physical and expression space, such that a small value indicates a cell located close to the root cell that also has a similar transcriptional profile. This remains a largely unexplored question and promising for future work, considering different ways for constructing such a combined spatial pseudotime beyond a simple linear combination and investigating potential inferences into the development of tissue and biological structures.

Similarly, multi-nomics integration, previously studied in scRNA-seq data, is posed for improvement in applications to spatial transcriptomics data. While multi-omics integration combining single-cell transcriptomics data with proteomics or other forms of data has been performed in the past, extending these methods to explicitly handle positional data would leapfrog on the additional inferences that spatial data provides. Recent research has begun to explore this, such as a study of fibroblast fate during tissue repair, integrating single cell chromatin landscapes (scATAC-seq), gene expression states (scRNA-seq), and spatial transcriptomic profiling^59^. Future developments in the collection of spatial -omics data will create a further need for integration of multiple fully spatial datasets.

New developments of research on these interesting problems, as well as many more that have yet to be discovered, place spatial transcriptomics in a position to create a revolution in the understanding of expression and behavior of cells even beyond that of single cell transcriptomics. Because of the additional dimension of complexity created by spatial data, we emphasize the need for a detailed understanding of the nature of different types of ST data and ST analyses, and how these aspects affect the ultimate conclusions.

## Supplementary information

Supplementary Information
