# Supplementary material for: Deciphering tissue structure and function using spatial transcriptomics
Source: Commun Biol. 2022 Mar 10;5:220. doi: 10.1038/s42003-022-03175-5 (PMC8913632; doi:10.1038/s42003-022-03175-5)
Supplement: Supplementary file 1 — Supplementary Information [file 42003_2022_3175_MOESM1_ESM.pdf]

Supplementary Note 1

Spatially-variable genes

Typically, methods for identifying SVGs seek to explicitly or implicitly model the expression of a gene *i* as a function of spatial position $g_i(\vec{X}): R^d \rightarrow R$, and then represent this function as a combination of a spatial term and spatially uniform random variation:

$$g_i(\vec{X}) = S(\vec{X}) + \epsilon$$

where $S(\vec{X})$ may represent a function or a random process, but designed in a way to depend on the spatial positions of cells. Under this framework, when the magnitude of $S(\vec{X})$ is significantly greater than that of ϵ, the function $g_i(\vec{X})$ is almost entirely determined by $S(\vec{X})$ and is therefore highly spatially correlated. On the other hand, if the magnitude of $S(\vec{X})$ is significantly less than that of ϵ, there is little spatial information in $g_i(\vec{X})$.

Segmentation

In general, segmentation can be viewed as an optimization problem over the space of all partitions $\mathcal{P}$ of N spots, each of which has a position $\overrightarrow{X_\iota}$ and an expression profile $\overrightarrow{g_\iota}$, into k groups such that each partition P is a vector

$$P = \vec{c} \in R^N \qquad c_i \in \{1,2,\dots,k\}$$

of the spatial locations to maximize some measure of homogeneity within each group. In the case of spatial data, this homogeneity should reflect both the transcriptional profiles and the positions, so that cells/spots that are nearby are likely to be in the same group. A potential formulation of the optimization problem for the solution partition P^* may take the form

$$P^* = \text{argmin}_{P \in \mathcal{P}} \alpha \sum_{i,j} \delta(c_i, c_j)\, d_g(\overrightarrow{g_\iota}, \overrightarrow{g_J}) + \beta \sum_{i,j \text{ adjacent}} \left(1 - \delta(c_i, c_j)\right)$$

where d_g is a measure of distance in gene expression and space, and $\delta(c_i, c_j)$ represents the Dirac delta function, equal to 1 when $c_i = c_j$ and 0 otherwise. In this case the first term encourages similar gene expression across spots in the same community, and the second term encourages adjacent spots to be in the same community. The specific formulation of the objective will vary from method to method.

Deconvolution analysis

Given an expression vector for a spot $\vec{g}_S$ and a set of k single-cell expression vectors $\vec{m}_i, i = 1, \ldots, k$ encoding the same set of genes, the cell type decomposition can be expressed as

$$\vec{g}_s = \sum_{i=1}^{k} c_i \vec{m}_i + \vec{r} + \text{constraints}$$

where the coefficients $c_i \geq 0$ represent the contribution of each single cell expression vector and $\vec{r}$ is a residual vector to be minimized. Possible sources of sets of vectors $\vec{m}_i$ may be expression patterns of known cell types, clustering of scRNA-seq data, or directly from individual cells in scRNA-seq data. The key requirement is that each vector be representative of a possible observation of expression in a single cell, as opposed to spots whose combined expression may not be similar to that of any single cell. Additional constraints reflect the fact that each spot is comprised of only a small number of cells – for this reason, typically k is constrained to not be large, and the coefficients c_i are constrained to not be too small. Algorithms may choose to truncate small coefficients or force representations with a fixed number of cells (e.g. doublets) to address this.
